# MV1035 Overcomes Temozolomide Resistance in Patient-Derived Glioblastoma Stem Cell Lines

**DOI:** 10.3390/biology11010070

**Published:** 2022-01-04

**Authors:** Alessio Malacrida, Alessandro Di Domizio, Angela Bentivegna, Giacomo Cislaghi, Eleonora Messuti, Silvia Maria Tabano, Carlo Giussani, Valentina Zuliani, Mirko Rivara, Gabriella Nicolini

**Affiliations:** 1 School of Medicine and Surgery, University of Milano-Bicocca, Via Cadore 48, 20900 Monza, Italy; alessio.malacrida@unimib.it (A.M.); angela.bentivegna@unimib.it (A.B.); e.messuti@campus.unimib.it (E.M.); carlo.giussani@unimib.it (C.G.); gabriella.nicolini@unimib.it (G.N.); 2 Milan Center for Neuroscience, University of Milano-Bicocca, Piazza dell’Ateneo Nuovo 1, 20126 Milan, Italy; 3 SPILLOproject, Via Stradivari 17, 20037 Paderno Dugnano, Italy; alessandro.didomizio@spilloproject.com (A.D.D.); giacomo.cislaghi@spilloproject.com (G.C.); 4 Inter-University Center for the Promotion of the 3Rs Principles in Teaching & Research (Centro 3R), 56122 Pisa, Italy; 5 Laboratory of Medical Genetics, IRCCS Ca’ Granda, Ospedale Maggiore Policlinico, Via Francesco Sforza 35, 20122 Milan, Italy; silvia.tabano@unimi.it; 6 Department of Pathophysiology and Transplantation, Università degli Studi di Milano, Via Francesco Sforza 35, 20122 Milan, Italy; 7 Neurosurgery Unit, Department of Neuroscience, S. Gerardo Hospital, 20900 Monza, Italy; 8 Food and Drug Department, University of Parma, Parco Area delle Scienze 27/A, 43124 Parma, Italy; valentina.zuliani@unipr.it

**Keywords:** glioblastoma, temozolomide, patient-derived GSCs, ALKBH2

## Abstract

**Simple Summary:**

Since 2005, temozolomide (TMZ) has been used as a standard first-line treatment for glioblastoma (GBM, grade IV glioma), and despite many studies and efforts no better alternatives have emerged. Tumor recurrences and TMZ resistance are common and the prognosis is very poor with a median overall survival of 14–16 months. The development of new pharmacological strategies is even more difficult due to the presence of glioma stem cells (GSCs). In this multidisciplinary study, we tested the imidazobenzoxazin-5-thione MV1035, alone and in combination with TMZ, in U87-MG and patient-derived (PD) GSCs in order to demonstrate a putative synergic effect. MV1035 was tested following its in silico predicted ability to act as an inhibitor against ALKBH2 and ALKBH5, both involved in maintaining the tumorigenicity of glioblastoma.

**Abstract:**

Glioblastoma (GBM, grade IV glioma) represents the most aggressive brain tumor and patients with GBM have a poor prognosis. Until now surgical resection followed by radiotherapy and temozolomide (TMZ) treatment represents the standard strategy for GBM. We showed that the imidazobenzoxazin-5-thione MV1035 is able to significantly reduce GBM U87-MG cells migration and invasiveness through inhibition of the RNA demethylase ALKBH5. In this work, we focus on the DNA repair protein ALKBH2, a further MV1035 target resulting from SPILLO-PBSS proteome-wide scale in silico analysis. Our data demonstrate that MV1035 inhibits the activity of ALKBH2, known to be involved in GBM TMZ resistance. MV1035 was used on both U87-MG and two patient-derived (PD) glioma stem cells (GSCs): in combination with TMZ, it has a significant synergistic effect in reducing cell viability and sphere formation. Moreover, MV1035 induces a reduction in MGMT expression in PD-GSCs cell lines most likely through a mechanism that acts on MGMT promoter methylation. Taken together our data show that MV1035 could act as an inhibitor potentially helpful to overcome TMZ resistance and able to reduce GBM migration and invasiveness.

## 1. Introduction

Glioblastoma multiforme (GBM) is the most aggressive and malignant primary brain tumor, classified as WHO (World Health Organization) grade IV gliomas [1]. GBM accounts for 69% of all gliomas and for 12–15% of all primary brain tumors [2]. Patients with GBM have a poor median overall survival (OS) of 14–16 months from diagnosis with gross total surgical resection and adjuvant chemo-radiation therapy. Nowadays, there are no effective treatments and tumor recurrence is expected. After the introduction of the alkylating agent temozolomide (TMZ) in 2005 as a standard first-line treatment, no other improvement has been achieved [3,4]. Moreover, so far, there is no standardized second-line treatment after tumor recurrence [5]. Multiple factors contribute to the treatment-refractory nature of glioblastoma including infiltration of normal brain, limited drug delivery into regions of tumor with an intact blood–brain barrier (BBB), inter- and intra-tumoral molecular heterogeneity, and inherent chemo- and radio-resistance [6]. A robust gene expression-based molecular classification of GBM identified proneural, neural, classical, and mesenchymal subtypes [7], and single-cell RNA sequencing revealed that multiple subtypes can reside within the same tumor [8], confirming cellular heterogeneity. The marked intratumoral heterogeneity is due to the presence of different cell populations with distinct genetic mutations, differentiation status, and responses to external stimuli. These include tumor populations characterized by greater tumorigenic potential called glioma stem cells (GSCs). They are characterized by self-renewal, stem cell markers expression (e.g., CD133, Nestin), elevated invasive behavior, chemo and radiotherapy resistance, ability to generate multi-lineage progenitors, and are the source not only for tumor initiation but also for recurrence.

GBM alkylating drug resistance is largely due to damage repair mechanisms involving principally O6-methylguanine-DNA methyltransferase (MGMT) and alkylated DNA repair protein 2 (ALKBH2) [9]. In particular, MGMT removes TMZ-induced O6meG, reversing the drug cytotoxicity [10]. In many tumors, silencing or reduction of MGMT expression is due to CpG methylation of the MGMT promoter [11]. Among others, 34–45% of glioblastoma patients present methylated MGMT promoter [12,13] and it is well documented that epigenetic silencing of MGMT gene correlates with good sensitivity to TMZ treatment [14]. Although the MGMT pathway is the most involved in the repair of damage induced by alkylating agents, also ALKBH2 and ALKBH3 participate in removing 1-meA and 3-meA. ALKBH’s role in TMZ-resistance is less studied with respect to MGMT but Johannessen et al. demonstrated that ALKBH2 expression in human GBM is higher with respect to the non-tumoral human brain and that ALKBH2 level of expression correlates directly with TMZ resistance [15].

In this work, we further investigated the imidazobenzoxazin-5-thione MV1035, and its ability to significantly reduce GBM U87-MG cell migration and invasiveness through inhibition of the RNA demethylase ALKBH5, as previously demonstrated [16].

Here, we focus on ALKBH2 since it emerged as an additional potential target of the imidazobenzoxazin-5-thione MV1035 from SPILLO potential binding sites searcher (SPILLO-PBSS, https://www.spilloproject.com (last access 2 January 2022)) proteome-wide scale in silico analysis [16,17]. In fact, besides ALKBH5, ALKBH2 was also predicted as one of the top-ranked potential targets whose enzymatic activity could be inhibited by the direct interaction with MV1035.

Since targeting multiple pathways involved in cancer development with a single compound represents a well-investigated approach to develop new cancer treatments [18], we investigated MV1035 ability to inhibit both ALKBH5 and ALKBH2 aiming to find a new drug able to hamper GBM progression and/or support TMZ therapy; in fact, combination therapy, especially for the most difficult to treat cancers, remains a keystone in therapy. Working with a synergistic approach could lead to therapeutic improvements due, for example, to the possibility to use lower dosages of the single drugs and reduce the toxicity of the chemotherapy [19].

We aimed to demonstrate in vitro ability of MV1035 to act both alone or in combination with TMZ to inhibit cell viability not only in U87-MG cells but also in patient-derived (PD) glioma stem cells (GSCs) it is widely recognized how U87 can significantly diverge from human tumors and also to comply to the “3Rs principles” that promote the use of in silico and in vitro biological systems to select the most promising compounds to be tested in in vivo models allowing to limit the number of animals used.

Importantly, we demonstrate that in PD-GSCs, MV1035 has a synergistic effect with TMZ in reducing cell viability and their ability to form spheres. Moreover, we show that MV1035 is able both to reduce the expression of MGMT and to inhibit ALKBH2 activity. These data, together with our recently published data regarding MV1035 inhibition of ALKBH5 activity, provide a rationale to suggest MV1035 as a useful starting point to develop new compounds able to overcome TMZ resistance and to reduce GBM migration and invasiveness.

## 2. Materials and Methods

### 2.1. Protein Database Preparation

The preparation of the protein database used for SPILLO-PBSS screening has been already described [16].

In summary, the database included 14,537 3D holo- and apo-protein structures available from the RCSB Protein Data Bank [https://www.rcsb.org/] (update September 2017, last access 2 January 2022), experimentally solved by either X-ray diffraction or solution NMR, excluding 100% sequence identity redundancies.

### 2.2. RBS Generation

The procedure followed to generate the MV1035 reference binding site (RBS) has been already detailed in [16] and amino acids included in the RBS are reported in Supplementary Table S1.

In particular, the RBS included 15 amino acid residues directly interacting with MV1035, without any water-mediated contact. It was generated by molecular modeling techniques and following the protocol previously [16] described.

### 2.3. In Silico Screening and Ranking of the Protein Database

The protocol used to search for potential targets and potential binding sites (PBSs) of MV1035 within all 3D protein structures included in the database has been already reported [16].

A systematic and unbiased search for the MV1035 PBSs within all 3D protein structures included in the database was carried out by SPILLO-PBSS using the following parameters: grid spacing: 2.0 Å, rotation step: 30°, geometric tolerance: 5.5 Å. This allowed us to generate a ranking of all proteins in the database and highlight potential targets of the MV1035 molecule.

### 2.4. Synthesis of MV1035

MV1035 was synthesized and purified in accordance with the already published procedures [20,21].

In particular, a solution of 2-(4-methyl-5-propyl-1H-imidazol-2-yl) phenol (0.80 mmol) and 1,1′-thiocarbonyldiimidazole (TCDI; 2.00 mmol) in 2 mL THF was prepared in a sealed 10 mL vial and was irradiated with microwaves for 10 min, setting the temperature at 90 °C and the maximal power output at 240 W. The mixture obtained was further added of TCDI (1.00 mmol) and heated at 90 °C for an additional 10 min, with the maximal power output at 240 W. During this period, the reaction vessel was stirred and cooled (2 atm air). The solvent was then concentrated under reduced pressure and the desired product was isolated and purified on silica gel using methylene chloride/n-hexane (1/1). mp 98–100 °C. 1H NMR (300 MHz, DMSOd6): δ 8.09 (dd, 1H), 7.61 (m, 2H), 7.48 (t, 1H), 3.21 (t, 2H), 2.27 (s, 3H), 1.62 (m, 2H), 0.91 (t, 3H).

MV1035 was dissolved in DMSO to a 10 mM stock solution and then was diluted to each desired working concentration with the respective working solution.

### 2.5. Cell Free Assay

The activity of ALKBH2 was evaluated with a specific DNA demethylation assay. 0.6 µg/µL of active recombinant ALKBH2 protein (Active Motif, Carlsbad, CA, USA) were incubated with 1 µg/50 µL of a specific methylated ssDNA sequence (5′-AAAGCAG(1 mA)ATTCGAAAAAGCGAAA-3′; Ella Biotech, Fürstenfeldbruck, Germany). The reaction was carried out in a buffer (50 mM Hepes pH 8.0, 50 µM Fe(NH4)_2_(SO4)_2_, 1 mM 2-oxoglutarate, 2 mM ascorbic acid) with or without MV1035. In the negative control, ALKBH2 protein was not added to the reaction. After 30 min of incubation at 37 °C, the reaction was thermally inhibited at 95 °C for 5 min. Subsequently, the ssDNA resulting from the previous reaction was mixed with equimolar amounts (1 µg/50 µL) of the complementary and unmethylated ssDNA (3′-TTTCGTCTTAAGCTTTTTCGCTTT-5′). The annealing reaction between the two ssDNA sequences was performed in a thermal cycler with the following setup: 2 min at 95 °C, 45 min at 25 °C, 5 min at 4 °C. After annealing, DNA was digested with EcoRI restriction enzyme: DNA was incubated at 37 °C for 45 min with EcoRI buffer and EcoRI restriction enzyme, following manufacturer instruction (Thermofisher, Waltham, MA, USA). EcoRI was then thermally inhibited at 65 °C for 20 min. Digested and undigested DNA were run in native polyacrylamide gel. Finally, DNA was stained with SYBR safe and visualized under a UV lamp.

### 2.6. Glioma Cell Lines

We used U87 cell line and two patient-derived glioma stem cell lines (PD-GSCs), G179 extensively characterized [22,23], and GSC7, recently isolated and described in Giambra et al. [24].

The U87-MG cell line was cultured in DMEM low glucose medium, supplemented with 10% FBS, 1% L-glutamine and 1% penicillin and streptomycin (Euroclone S.p.A., Milan, Italy).

PD-GSCs were cultured in a selective medium for NSC composed by DMEM F-12 and Neurobasal 1:1, B-27 supplement without vitamin A (Life Technologies Italia, Milan, Italy), 2 mM L-glutamine (Euroclone S.p.A., Milan, Italy), 10 ng/mL recombinant human bFGF and 20 ng/mL recombinant human EGF (Miltenyi Biotec, Bergisch, Gladbach, Germany), 20 UI/mL penicillin and 20 μg/mL streptomycin (Euroclone S.p.A., Milan, Italy). After the isolation, the medium was replaced every 3 days to remove stroma and red blood cells residues, catabolic products and to supply fresh nutrients. Debris and adherent death cells generally were eliminated after a couple of passages. The isolated cells propagate in culture as free-floating spheres defined as tumorspheres [25], which appeared in 15–20 days of culture after isolation. When tumorspheres reached an average size of 100 μm in diameter, the culture was ready to be passed and expanded. At each passage (P), tumorspheres were mechanically dissociated using a sterilized p200 pipette set at 180–200 μL and pipetting up and down 100–150 times to achieve a single-cell suspension. Cells were incubated at 37 °C and 5% CO_2_ in a humidified incubator.

### 2.7. Cytotoxicity Assay

The evaluation of cell viability after MV1035, TMZ, and combinations between the two drugs was performed with the MTT assay. U87-MG, G179, and GSC7 cells were seeded in 96-well plates at 5 × 10^3^ cells/well density. Cells were then treated with different concentrations and combinations of MV1035 (10–50 µM) and TMZ (75–1200 µM). After 24 h of incubation, the MTT assay was performed. (3-(4,5-dimethylthiazol-2-yl)-2,5-diphenyltetrazolium bromide) MTT solution was directly added to the culture medium to reach a final concentration of 0.5 mg/mL. After 4 h, plates were centrifuged at 1000× *g* for 10 min, the supernatant was removed, and formazan crystals were solubilized in acidified 2-propanol. Absorbance was measured in a microplate reader at 570 nm (BMG Labtech, Ortenberg, Germany). The expected drug combination responses between MV1035 and TMZ were calculated based on the ZIP reference model using the SynergyFinder web application [26].

### 2.8. Limiting Dilution Assay (LDA)

GSCs were plated at 1, 5, 10, 20, 40, and 80 cells per well into a 96-well plate. After one day, cells were treated with DMSO, TMZ (200 μM), MV1035 (25 and 50 μM), or a combination of TMZ and MV1035. Seven days post treatment, phase-contrast images were obtained to visualize the morphology of the sphere and the number of neurospheres in each well was quantified by manual counting. Data were quantified by Extreme limiting dilution analysis (ELDA) software [27].

### 2.9. MGMT Expression

MGMT protein expression was evaluated by Western blotting. 250 × 10^3^ U87-MG, G179, and GSC7 cells were seeded and treated with MV1035. After 24 h, cells were chemically and mechanically lysed with RIPA buffer and cell scraper. Protein samples were then clarified by centrifugation (13,000× *g*, 15 min, 4 °C) and quantified using the Bradford method. Then, 10 µg of proteins was separated in a SDS-PAGE gel and transferred to a nitrocellulose membrane. Western blotting was performed following the antibodies manufacturer’s instructions (anti-MGMT, 1:1000, Novus Bio, Centennial, CO, USA; anti-actin, 1:1000, Santa Cruz, Dallas, TX, USA; anti-mouse, 1:2000, Chemicon, Temecula, CA, USA; anti-goat, 1:2000, Chemicon, Temecula, CA, USA).

### 2.10. MGMT Methylation Analysis

Pyrosequencing experiments were aimed to quantitatively evaluate the methylation levels of 10 CpG-sites for the MGMT gene. The primers used were the followings: MGMT: forward: 5′-GTTTYGGATATGTTGGGATAG-3′, reverse: 5′biotin-CRACCCAAACACTCACCAAA-3′, seq: 5′-GATAGTTYGYGTTTTTAGAA-3′.

PCRs were carried out using 20 ng of bisulphite-converted DNA from cell lines in a final volume of 50 uL, with 10 pmol forward and reverse primers, one of them being biotinylated. Quantitative DNA methylation analyses were performed using the Pyro Mark ID instrument in the PSQ HS 96 System (Biotage AB, Uppsala, Sweden), with the PyroGold SQA Reagent Kit (Biotage AB, Uppsala, Sweden) according to the manufacturer’s instructions. Raw data were analyzed using the Q-CpG software v1.0.9 (Biotage AB, Uppsala, Sweden), which calculates the ratio of converted C’s (T’s) to unconverted C’s at each CpG, giving the percentage of methylation. For each sample, the methylation value represents the mean between two independent PCR and pyrosequencing experiments.

## 3. Results

### 3.1. SPILLO-PBSS Screening

#### 3.1.1. Protein Database Ranking

**Figure 1:**
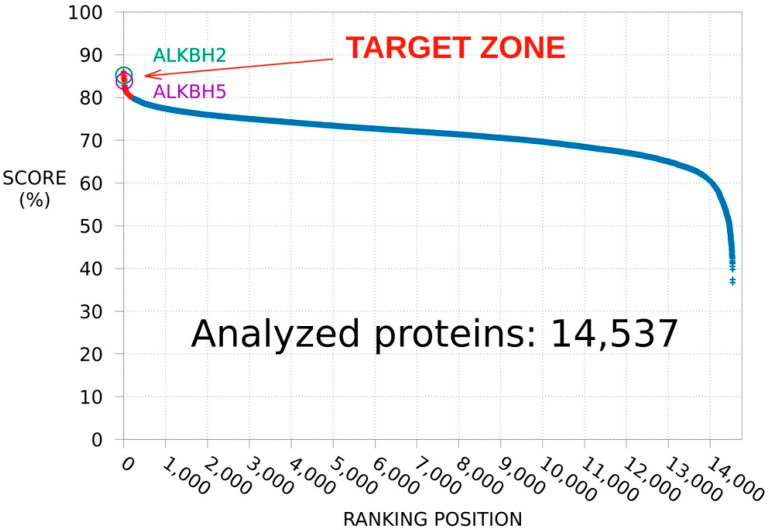
SPILLO-PBSS screening and ranking of the available human structural proteome—Plot resulting from the in silico screening and ranking carried out by SPILLO-PBSS on the available human structural proteome (14,537 protein structures retrieved from the RCSB Protein Data Bank [https://www.rcsb.org/] (last access 2 January 2022) in September 2017, excluding 100% sequence identity redundancies). ALKBH2 and ALKBH5 enzymes were found in the “Target zone” (in red, defined as the set of points that are on the left side of the point of maximum upward concavity of the curve) among the top-20 potential targets of MV1035.

With the aim of identifying MV1035 target proteins able to account for MV1035-induced reduction of U87-MG glioblastoma cells migration and invasiveness, we performed an analysis of the whole protein database using the SPILLO-PBSS software. Results are summarized by the plot in Figure 1, in which points correspond to proteins ranked in descending order according to the highest similarity between the reference binding site (RBS) and the best potential binding site (PBS) identified within each 3D protein structure. The potential targets of MV1035 are those with scores clearly higher than all others.

#### 3.1.2. ALKBH2 as a Further Potential Target of MV1035

Besides the RNA demethylase ALKBH5 (PDB code: 4OCT), and despite sharing <20% sequence identity with this protein (calculation performed by EMBOSS Needle, https://www.ebi.ac.uk/Tools/psa/emboss_needle/, last access 2 January 2022), the DNA oxidative demethylase ALKBH2 (PDB code: 3BU0) was one of the 20 top-ranked (out of 14,537) targets of MV1035 (6 of which involved in cancer) identified by our previous SPILLO-PBSS analysis [16]. Both ALKBH5 and ALKBH2 are highly expressed in glioblastoma and this is the reason why we started our experimental validation from these two proteins, while an in-depth analysis and validations for the other top-ranked potential targets will be the matter of future studies. As described in the next paragraph, we were able to hypothesize an inhibition of the catalytic activity of ALKBH2 by MV1035. Overall, these findings prompted us to experimentally test the SPILLO-PBSS-predicted interaction between ALKBH2 and MV1035, which could make this molecule even more interesting because of its polypharmacological therapeutic profile.

#### 3.1.3. Competitive Inhibition Hypothesis

**Figure 2:**
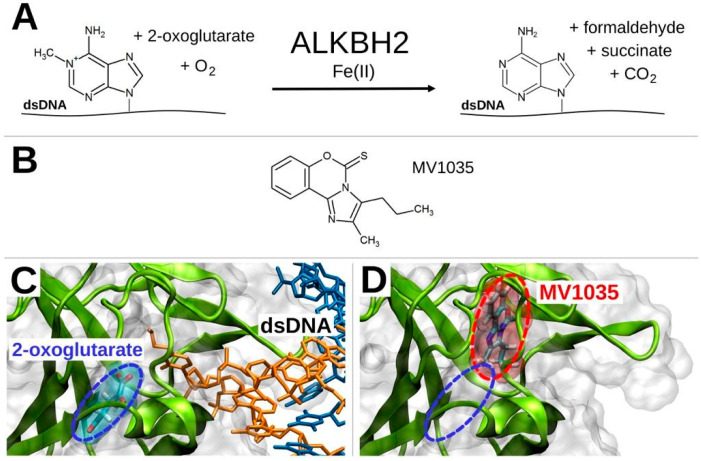
ALKBH2 enzymatic reaction and competitive inhibition hypothesis. The enzymatic reaction (**A**) catalyzed by ALKBH2 is reported along with the 2D formula of MV1035 (**B**). The positions of 2-oxoglutarate (**C**) and MV1035 (**D**) within their corresponding binding sites are also reported, as obtained by X-ray diffraction (PDB code: 3BU0) and SPILLO-PBSS calculation, respectively. The partial overlapping between the binding sites of 2-oxoglutarate and MV1035 is shown, which implies a competition between the two molecules for the same binding region that leads to an inhibition of the catalytic activity of ALKBH2 (drawings rendered using VMD [30]).

As for ALKBH5, also for ALKBH2 a PBS for MV1035 was identified within the catalytic site of the enzyme (see Supplementary Table S1) where the conversion of 1-methyladenosine (1-meA) to adenosine in dsDNA, in the presence of 2-oxoglutarate, molecular oxygen, and iron(II) takes place (Figure 2A). Importantly, as demonstrated for ALKBH5, also in this case the PBS was partially overlapped with the region occupied by 2-oxoglutarate and this allowed us to hypothesize an inhibition of the catalytic activity of the enzyme by MV1035 through the competition with 2-oxoglutarate for the same binding region. It may be expected that the presence of MV1035 makes it difficult for the methylated nucleobase within DNA to reach the right site for the enzymatic reaction (Figure 2C,D). Noteworthy, the PBS for MV1035 within ALKBH2 was identified despite being partially closed and apparently inaccessible to the ligand (see Supplementary Figure S1 and Supplementary Table S2) thanks to SPILLO-PBSS’s ability to take into account protein flexibility [16,28,29].

### 3.2. Target Validation

#### ALKBH2 Inhibition by MV1035: Cell Free Assay

**Figure 3:**
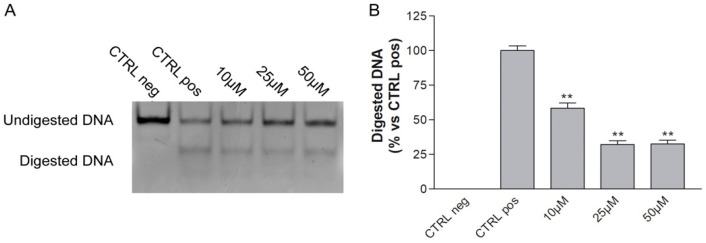
Representative image (**A**) and quantification graph (**B**) of ALKBH2 cell-free activity assay. Negative CTRL without ALKBH2 protein, positive CTRL with ALKBH2 protein and without treatments, increasing concentrations of MV1035 (10, 25, and 50 µM). Data are represented as the mean percentage ± SD compared to untreated controls, arbitrarily set to 100%. ** *p* < 0.01 vs. CTRL.

A cell-free ALKBH2 activity assay was performed to evaluate the SPILLO-PBSS predicted inhibitory potential of MV1035. Methylated ssDNA oligonucleotide was incubated in the presence or absence of ALKBH2 and in the presence or absence of treatment with MV1035. After annealing with a complementary ssDNA oligonucleotide, DNA was digested with the *Eco*RI restriction enzyme. In negative CTRL, as expected due to the absence of ALKBH2, all the DNA is undigested, as it remains methylated and therefore not recognizable by the *Eco*RI restriction enzyme. On the contrary, in positive CTRL, the DNA is partially digested, while the treatment with MV1035 induces a statistically significant inhibition at every dose tested (Figure 3).

### 3.3. Biological Validation

#### 3.3.1. Cytotoxicity Assay

Temozolomide, since 2005, represents the standard first-line treatment for GBM [3,4].

In order to evaluate the effect of MV1035 and TMZ against the U87-MG glioblastoma cell line and two different PD-GSC lines (G179 and GSC7) an MTT assay has been performed. Cells were treated with concentrations of MV1035 (10, 25, and 50 µM) previously tested for their ability to inhibit ALKBH2 and different TMZ concentrations. Since several TMZ IC50s are reported in the literature against glioblastoma cells [31,32], we have decided to treat our cells with a wide range of concentrations (75, 150, 300, 600, 1200 µM), alone or in combination with MV1035, for 24 h.

**Figure 4:**
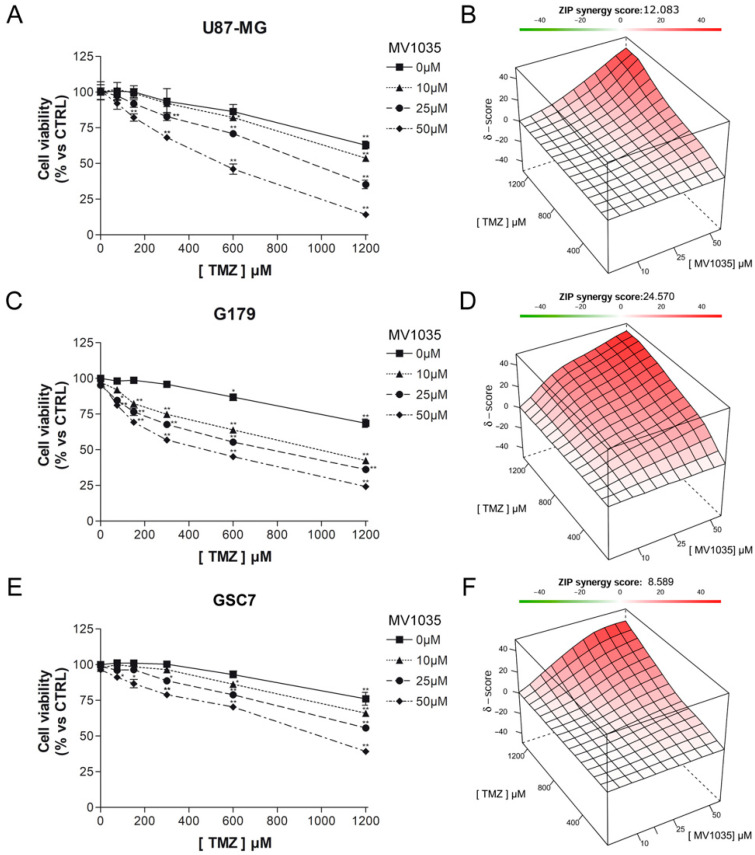
MTT assay of U87-MG (**A**), G179 (**C**) and GSC7 (**E**) cells treated for 24 h with combinations of different concentrations of MV1035 (10, 25 and 50 µM) and TMZ (75, 150, 300, 600, 1200 µM). Data are represented as the mean percentage ± SD compared to untreated controls, arbitrarily set to 100%. Synergism analysis between MV1035 and TMZ in U87-MG (**B**), G179 (**D**), and GSC7 (**F**) cells. * *p* < 0.05, ** *p* < 0.01 vs. CTRL.

No concentrations of MV1035 evaluated are able to reduce cell viability of GBM cells and the results are comparable to untreated controls. TMZ reduces the cell viability of all GBM cells in a dose-dependent manner (Figure 4). Its IC50 values were 1578 µM for U87-MG, 1984 µM for G179, and 1951 µM for GSC7 (Table 1).

The combination between MV1035 and TMZ is significantly synergic. The simultaneous treatment with the two molecules reduces the cell viability of all considered cell lines more than single treatments. The highest synergism is achieved by the two highest concentrations of MV1035 and TMZ (Figure 4). The IC50 of TMZ significantly decreases when used in combination with MV1035 (Table 1).

#### 3.3.2. Limiting Dilution Assay

**Figure 5:**
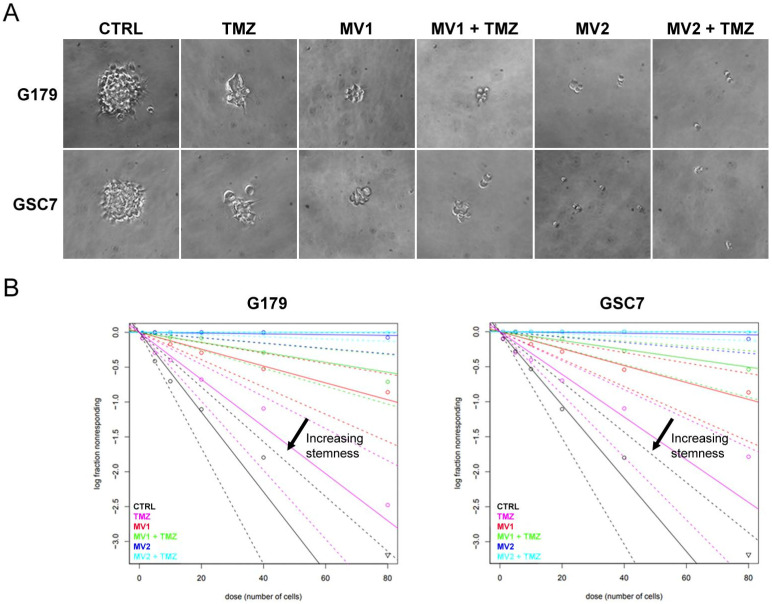
In vitro analysis of neurospheres formation using the limiting dilution assay (LDA). (**A**) representative images of neurospheres in G179 and GSC7 following 7 days of TMZ (200 µM) and/or MV1035 (MV1 25 µM; MV2 50 µM) treatments. Images were captured with Leica DFC290 microscope camera with 20× magnification. (**B**) LDA data analysis by the extreme limited dilution assay (ELDA) tool. The amount of initially seeded cells (*x* axis) is plotted against the log fraction of non-responders corresponding to wells without any detected sphere (*y* axis). The slope of the line represents the log-active cell fraction. The dotted lines give the 95% confidence interval.

The ability to form neurospheres is a characteristic of GSCs [25]. In order to study the possible MV1035 inhibitor effect on self-renewal ability, we performed a sphere limiting dilution assay followed by quantification with ELDA Tool. We analyzed the effect of 25 and 50 µM MV1035 (concentrations showing higher synergic effect with TMZ in the MTT test) and 200 µM TMZ (the highest concentration ineffective in reducing PD cell lines vitality), alone and in combination, on the two PD GSC cell lines (G179 and GSC7). MV1035 alone, already at the lowest dose, leads to drastic inhibition in sphere number formation in both GSC lines; the contribution of TMZ is not appreciable in the combined treatment, confirming the data with MV1035 alone (Figure 5).

#### 3.3.3. MGMT Expression

The protein level of MGMT was found to be inversely related to the chemosensitivity of gliomas to alkylating agents [13,14].

In order to investigate a possible MV1035 effect on MGMT protein expression, Western blotting was performed on U87-MG, G179, and GSC7 cells before and after treatment with 50 µM MV1035 (concentration more effective in inhibiting PD neurosphere formation) for 24 h.

**Figure 6:**
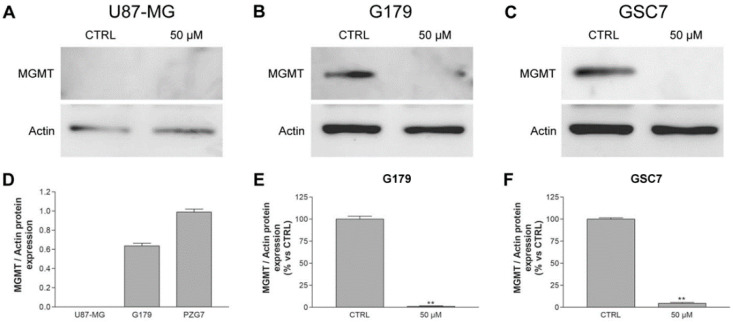
Representative images and quantification graphs of Western blots of MGMT in U87-MG (**A**), G179 (**B**,**E**), and GSC7 (**C**,**F**) cells, treated with MV1035 50 µM or not treated (CTRL) for 24 h. (**D**) Comparison of MGMT expression in untreated cells. Data are represented as the mean ± SD compared to untreated controls, arbitrarily set to 100%. ** *p* < 0.01 vs. CTRL.

U87-MG cells do not express MGMT protein and treatment with MV1035 does not induce MGMT protein expression (Figure 6A). Conversely, untreated G179 and especially GSC7 cells expressed MGMT. The treatment with MV1035 induced an almost total reduction of MGMT protein expression in both PD cell lines (Figure 6).

#### 3.3.4. MGMT Promoter Methylation

**Figure 7:**
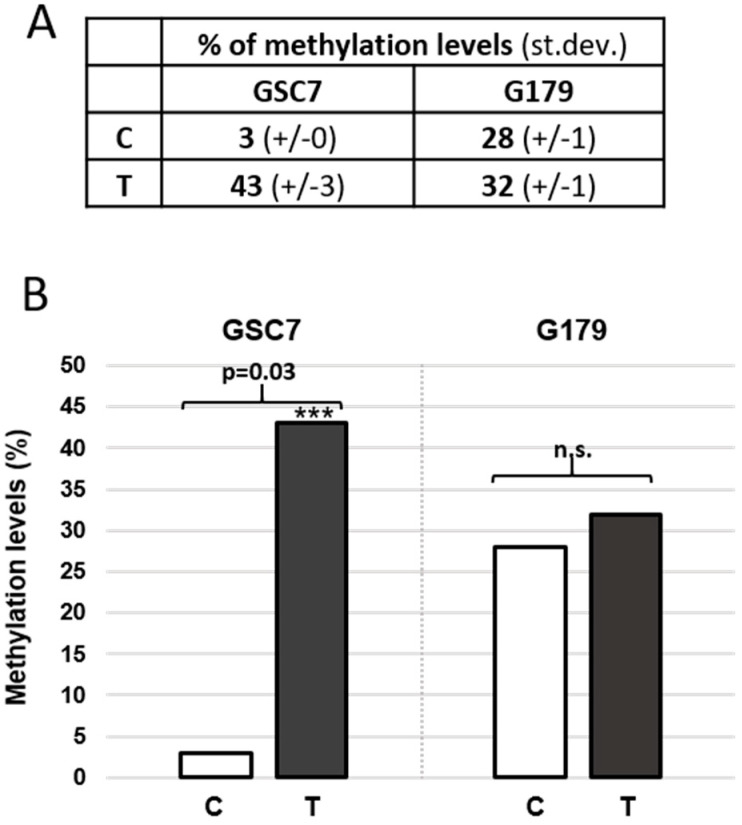
(**A**) MGMT promoter mean methylation levels (+/− standard deviation) in untreated (C) and treated (T) GSC7 and G179 cell lines. (**B**) Graphical representation of mean methylation levels in C and T samples of GSC7 and G179 cell lines: MGMT methylation significantly increased in GSC7, whereas was unchanged in G179 cells. *** *p* < 0.05. n.s.: not significative.

The reduced expression of MGMT after treatment with MV1035 may result from increased methylation of the gene promoter. In order to verify if the treatment with MV1035 is able to modify the methylation of the MGMT promoter, a quantitative CpG methylation analysis was performed by pyrosequencing after treatment with MV1035 50 µM for 24 h. As expected, the promoter of the U87-MG cell line, which does not express the MGMT protein (Figure 6A,D), does not undergo modifications after treatment (data not shown). As shown in Figure 7, MGMT methylation levels of the GSC7 cell line were significantly increased in treated vs. untreated samples, showing 43% and 3% of methylation, respectively (*p* = 0.03). Conversely, MGMT methylation levels of the G179 cell line were similar in treated vs. untreated samples, showing 32% and 28% of methylation, respectively (*p* = n.s).

## 4. Discussion

The design of strategies to overcome resistance to TMZ represents an important milestone for the treatment of glioblastoma. In fact, TMZ anticancer effect is highly inhibited by DNA repair mechanisms. Once metabolized, TMZ is converted in the active form which determines methyl adducts in nitrogen and oxygen atoms, inducing DNA alkylation. DNA direct reversal repair happens thanks to MGMT and Alkbh homologs ALKBH2 and ALKBH3.

Dual or multi-targeting of pathways involved in cancer development and progression by a single molecule represents a well-investigated approach for developing new cancer treatments [18]. Even if dual targeting could be seen as an alternative to combination therapy, the latter remains a cornerstone in battling cancer [19].

In the present work, using a multidisciplinary approach, we have deeply investigated the in vitro effect of the imidazobenzoxazin-5-thione MV1035 on glioblastoma aiming to demonstrate its capability to inhibit both ALKBH5 and ALKBH2 and its useful combination with TMZ as an effective approach to hinder glioma stem cell proliferation. In a previous paper [16] we have already demonstrated that MV1035 is able to significantly reduce U87-MG cell line migration and invasiveness inhibiting the RNA demethylase ALKBH5. Starting from the whole available human structural protein database (September 2017) SPILLO-PBSS screening and analysis, we have focused on the DNA repair protein AlkB 2 (ALKBH2), recognized by SPILLO-PBSS as a further MV1035 target. In particular, we hypothesized a reduction of the catalytic activity of the enzyme resulting from the competition between MV1035 and the natural substrates of ALKBH2 for the same binding region. Our cell-free analysis demonstrates that MV1035 directly inhibits active recombinant ALKBH2 protein and consequently methylation status of ssDNA oligonucleotide increases, further validating SPILLO-PBSS in silico prediction. Such results further confirm SPILLO-PBSS’s great potentialities in identifying targets and off-targets of any small molecule on a proteome-wide scale through a direct identification of their binding sites. Importantly, the inclusion of protein flexibility in the model allows not just the analysis of known binding sites but also the identification of totally unknown binding sites, that can be identified even when they are not already in a suitable conformation for the binding event, as in the case of the crystal structures of ALKBH5 and ALKBH2, where pockets were unsuitably arranged and sterically hindered. The capabilities of this innovative approach can attract great interest for its manifold applications including polypharmacological analysis, side effect prediction/clarification, and drug rescuing and repurposing.

ALKBH2 damage repair mechanism is involved in TMZ resistance [10] together with the alkyltransferase O6-methylguanine-DNA methyltransferase (MGMT).

Although MGMT is the best-known factor inducing TMZ resistance in GBM only half of the patients express the protein and 43–47.5% of patients present MGMT promoter methylation silenced [33]. Interestingly, several studies have demonstrated that MGMT expression is not related to gene deletion or mutation, or unstable RNA but principally to methylation of the CpG island of its promoter. Despite the methylation status of MGMT promoter results strictly related to GBM alkylating agents’ sensibility, the prognostic value of this parameter is still controversial [34,35].

On the other hand, ALKBH2 is highly expressed in human glioblastoma and in established GBM cell lines. ALKBH2 overexpression enhances GBM TMZ resistance whereas ALKBH2 silencing increases GBM TMZ sensitivity [36]. Lee et al. have also demonstrated that ALKBH2 expression is correlated in U87-MG resistance to photodynamic therapy (PDT).

Further aware that traditional, commercially available glioma cell lines, as U87-MG, maintained in conventional cell culture for long periods can diverge genetically from human tumors [37], limiting their translational utility, we investigated the MV1035 effect also on two different primary patient-derived (PD) glioma stem cell (GSC) lines, one of which previously characterized in our laboratory [24]. Moreover, several studies have demonstrated that primary patient-derived (PD)-GSCs, cultured in serum-free neurobasal medium, supplemented with epidermal growth factor (EGF) and fibroblast growth factor (FGF), are more representative of the tumor than traditional, commercially available GBM cell lines [22,38,39]. In fact, analysis carried out on the two PD-GSC lines, allows us to study the effect of MV1035 on the subpopulation of cells recognized to be responsible for resistance to pharmacological/radiation treatment and relapses. Interestingly the GSC7 line has been isolated from a biopsy which was molecularly characterized and found to have an unmethylated MGMT promoter (to date, no data about this parameter for G179 cells is present in literature). On the other hand, several studies have demonstrated that CD133+ GSCs express high levels of MGMT [40,41] and that differentiation of these cells is related to the reduction in MGMT expression and consequently to an increase in sensitivity to TMZ [42,43].

Our in vitro results suggest that strategies to inhibit the activity of ALKBH2, together with that of MGMT, are fundamental to counteract TMZ resistance.

In fact, we demonstrate that the treatment with MV1035 and TMZ has a synergistic effect both in reducing bidimensional cell culture GBM proliferation and PD-GSCs neurospheres formation. It is noteworthy that MV1035 at the higher concentration tested (50 μM) is able, alone, to totally inhibit PD-GSCs neurospheres formation. Moreover, MV1035 induced a reduction in MGMT expression in both PD cell lines G179 and GSC7. This data suggests that ALKBH2 demethylase activity could also modify MGMT promoter methylation status and consequently MGMT protein expression. This mechanism is confirmed by promoter methylation analysis in the GSC7 cell line. The behavior of the G179 cell line remains to be clarified. In fact, this cell line presents a higher basal methylation level of MGMT promoter than the GSC7 cell line which is not modified after MV1035 treatment; however, the protein level of G179 is significantly reduced after MV1035 treatment. Other mechanisms of regulation of MGMT expression need to be studied to understand this behavior, as for example di-methylation of histone H3K9, degradation of MGMT mRNA by microRNAs, or interference with protein translation by miR-648 [44].

The result in the GSC7 cell line is of great importance since it is widely recognized that the most effective strategy to hamper MGMT activity is to facilitate the methylation of its promoter [45]. A strategy that involved the pretreatment with the MGMT pseudo-substrate O6-benzylguanine (O6-BG) even if it proved effective in overcoming TMZ resistance, in a subsequent phase II trial showed a positive response in only one out of thirty-four patients with recurrent TMZ-resistant malignant glioma [46,47]. Moreover, studies focusing on the inhibition of MGMT using interferon (IFN)-b [48], levetiracetam [49], or a STAT3 inhibitor [50], never went beyond preclinical studies.

MV1035 is able to modify the methylation of the MGMT promoter, through the inhibition of ALKBH2. This, along with its already demonstrated ability to inhibit ALKBH5, makes MV1035 a very promising and strong candidate to be further developed. In fact, we demonstrated the in vitro ability of MV1035 to act both alone or in combination with TMZ to inhibit cell viability of different PD-GSCs before pushing the compound to the in vivo testing seeking to comply with the “3Rs principles” that aim to replace animal employment as long as possible with in silico and in vitro biological systems.

## 5. Conclusions

In this study, we demonstrate that MV1035 is able to induce a reduction in MGMT expression showing a synergic effect if combined with TMZ on both cell line UG87-MG cell line and especially on PD-GSCs cell lines. Moreover, MV1035 modifies the methylation status of the MGMT promoter in the GSC7 cell line. Consequently, this compound appears to be a great candidate to be further developed starting from its ability to inhibit both ALKBH2 and ALKBH5 and following the good results obtained when used in combination with TMZ.

## Figures and Tables

**Table 1:** TMZ IC50 concentrations of different combinations between MV1035 and TMZ against U87-MG, G179, and GSC7 cells.

TMZ IC50 µM	MV1035			
	0 µM	10 µM	25 µM	50 µM
U87-MG	1578 ± 44	1295 ± 31	941 ± 79	465 ± 26
G179	1984 ± 198	1770 ± 30	1289 ± 112	869 ± 36
GSC7	1951 ± 142	988 ± 82	631 ± 36	357 ± 9

## Data Availability

The data presented in this study are available in the article and supplementary material.

## Supplementary Materials

The following are available online at https://www.mdpi.com/article/10.3390/biology11010070/s1, Figure S1: PBS for MV1035 within ALKBH2, Table S1: Amino acid residues included in the reference binding site (RBS) of MV1035 listed according to their relative stabilizing contribution to the ligand binding. Table S2: Amino acid residues of ALKBH2 (PDB code: 3BU0) overlapping with MV1035 in the potential binding site (PBS) identified by SPILLO-PBSS.
