# Supplementary material for: MV1035 Overcomes Temozolomide Resistance in Patient-Derived Glioblastoma Stem Cell Lines
Source: Biology (Basel). 2022 Jan 4;11(1):70. doi: 10.3390/biology11010070 (PMC8772739; doi:10.3390/biology11010070)
Supplement: Supplementary file 1 [file biology-11-00070-s001.zip › biology-1476883-supplementary.pdf]

Supplementary Information

MV1035 OVERCOMES TEMOZOLOMIDE RESISTANCE IN PATIENT-DERIVED GLIOBLASTOMA CELL LINES

Supplementary Table S1. Amino acid residues included in the reference binding site (RBS) of MV1035 listed according to their relative stabilizing contribution to the ligand binding. The corresponding amino acid residues of the best potential binding site (PBS) for MV1035 in ALKBH2 (PDB code: 3BU0) are also reported, as identified by SPILLO-PBSS.

**RBS**		**Best PBS in ALKBH2**
**Relative stabilizing contribution (%)**	**RBS residues**	**PBS residues**
25.81	ARG A 1	ARG A 254
15.20	TYR A 2	TYR A 122
15.02	SER A 3	THR A 252
12.76	PHE A 4	PHE A 124
10.66	LYS A 5	LEU A 127
3.61	ILE A 6	VAL A 108
3.02	LEU A 7	ILE A 168
2.45	ILE A 8	VAL A 99
2.08	VAL A 9	LEU A 157
1.86	VAL A 10	THR A 121
1.83	VAL A 11	ASN A 159
1.55	MET A 12	HIS A 236
1.54	ILE A 13	LEU A 129
1.52	LEU A 14	ILE A 256
1.09	LEU A 15	ARG A 110

Supplementary Table S2. Amino acid residues of ALKBH2 (PDB code: 3BU0) overlapping with MV1035 in the potential binding site (PBS) identified by SPILLO-PBSS. For each of them, the number of atoms involved in steric clashes (35 steric clashes in total) are also reported.

Amino acids of ALKBH2 overlapping with MV1035 in the PBS	Number of atoms overlapping with MV1035 in the PBS
ARG 110	6
GLN 112	5
TYR 122	7
THR 123	4
PHE 124	9
LEU 127	2
LEU 157	1
ASN 159	1

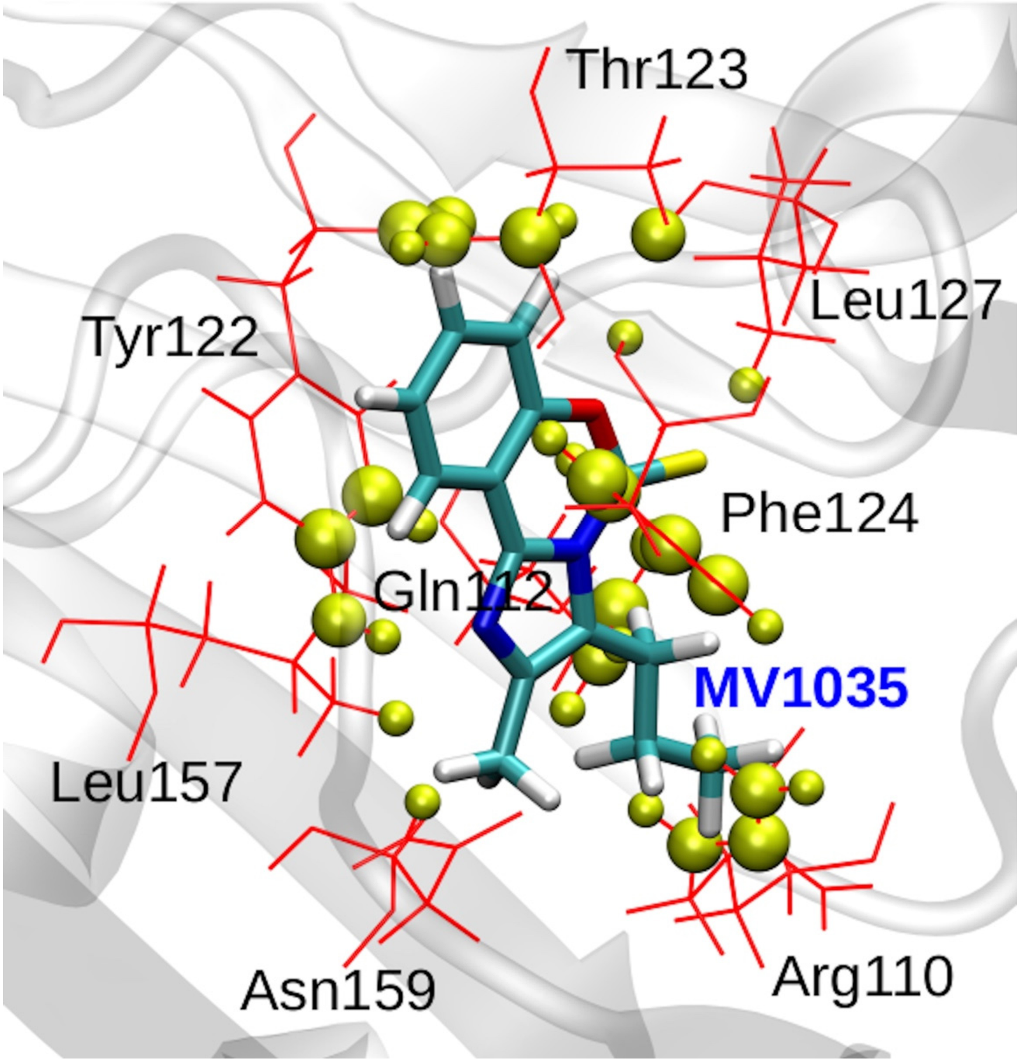

Supplementary Figure S1. Steric clashes between ALKBH2 (PDB code: 3BU0) and MV1035 present in SPILLO-PBSS output. The amino acids listed in Supplementary Table S2 and their atoms giving rise to steric clashes are reported in red and yellow, respectively. A distance of 2.0 Angs was here used as threshold below which the nuclei of any two atoms (belonging one to ALKBH2 and the other to MV1035) give rise to a steric clash.
