# Supplementary material for: MV1035 Overcomes Temozolomide Resistance in Patient-Derived Glioblastoma Stem Cell Lines
Source: Biology (Basel). 2022 Jan 4;11(1):70. doi: 10.3390/biology11010070 (PMC8772739; doi:10.3390/biology11010070)

MV1035 OVERCOMES TEMOZOLOMIDE RESISTANCE IN PATIENT-DERIVED GLIOBLASTOMA STEM CELL LINES

Figure 3

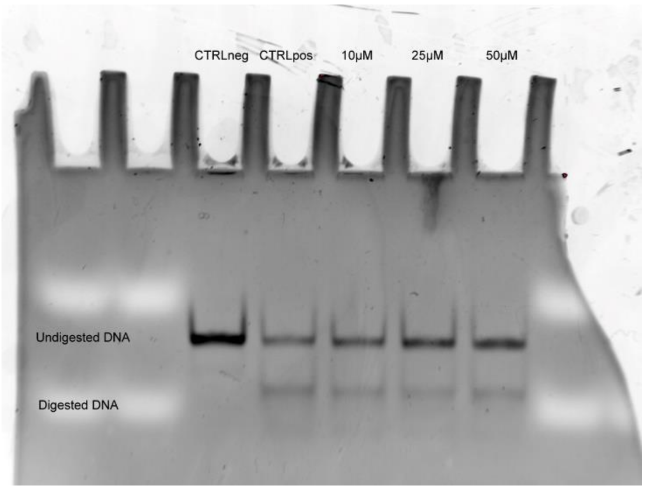

Figure 6

MGMT western blot

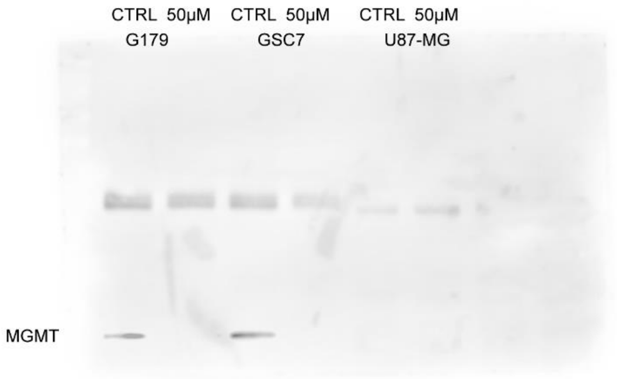

Actin western blot

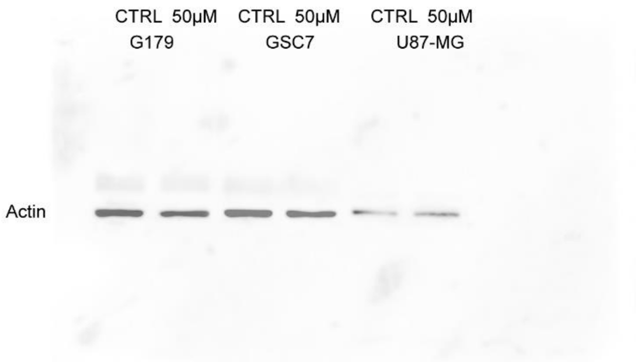

Supplement: Supplementary file 1 [file biology-11-00070-s001.zip › biology-1476883-WB.pdf]
